# The bridging role of gut microbiota-derived metabolites in neuropathic pain comorbid with anxiety

**DOI:** 10.3389/fnins.2026.1752839

**Published:** 2026-02-12

**Authors:** Jing Bian, Jinzhu Bai

**Affiliations:** 1 School of Rehabilitation Medicine, Shandong University of Traditional Chinese Medicine, Jinan, China; 2 School of Rehabilitation Medicine, Capital Medical University, Beijing, China; 3 Department of Pain Medicine, Beijing Bo’ai Hospital, China Rehabilitation Research Center, Beijing, China

**Keywords:** anxiety, gut microbiota, gut-brain axis, metabolites, neuropathic pain

## Abstract

Neuropathic pain (NP) is a chronic pain condition caused by damage or disease of the somatosensory system and often forms a comorbid state with anxiety, severely affecting patients’ quality of life. The occurrence of this comorbidity involves the interplay of multiple mechanisms, including neuroinflammation, metabolic abnormalities, the hypothalamic-pituitary-adrenal (HPA) axis dysregulation, and imbalances in central neurotransmitter systems. In recent years, research on the mechanisms by which gut microbiota-derived metabolites regulate NP and anxiety via the “gut-brain axis” has garnered increasing attention. Among the numerous gut microbiota-derived metabolites, lipopolysaccharide (LPS), short-chain fatty acids (SCFAs), bile acids (BAs), serotonin (5-HT), and γ-aminobutyric acid (GABA) are considered key signaling molecules. They collectively participate in the pathological process of NP-anxiety comorbidity by regulating immune responses, metabolic pathways, and neural pathways. This review focuses on these five metabolites, analyzing the bridging role of their functional abnormalities in this comorbidity and future directions in this field.

## 1 Introduction

Neuropathic pain (NP) is chronic pain caused by a lesion or disease of the somatosensory nervous system, with a global adult prevalence of 6.9%–10% (van Hecke et al., 2014). Clinical observations indicate that approximately 45% of chronic pain patients concurrently experience anxiety symptoms. This pain-anxiety comorbidity not only exacerbates the subjective suffering of patients but also leads to a multidimensional decline in their quality of life (Battaglia et al., 2020). Although current clinical management primarily involves polypharmacy with analgesics, antidepressants, and anxiolytics, it is often plagued by incomplete symptom relief, high recurrence rates, and adverse drug effects (Finnerup et al., 2015). Essentially, this comorbidity involves complex interactions among multiple pathophysiological processes, including central sensitization, neuroinflammation, and altered synaptic plasticity. The core molecular mechanisms remain incompletely understood, directly contributing to the limited clinical efficacy of existing treatments (Attal et al., 2023; Colloca et al., 2017). Recent studies have revealed that gut microbiota metabolites regulate neuroinflammation and neurotransmitter balance via the gut-brain axis (Yang and Cong, 2021), offering a new direction for research into the mechanisms and treatment of NP-anxiety comorbidity.

**FIGURE 1:**
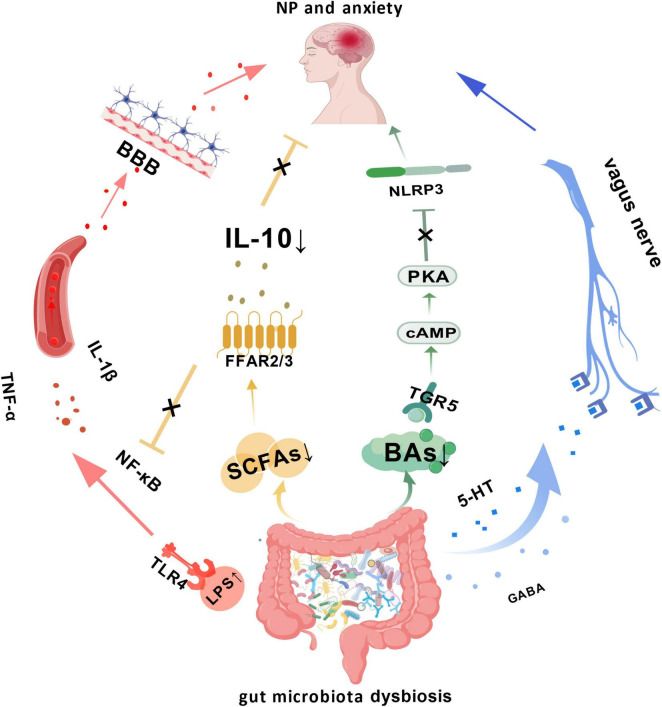
Schematic diagram illustrating how gut-derived lipopolysaccharide (LPS), short-chain fatty acids (SCFAs), bile acids (BAs), serotonin (5-HT),1 and γ-aminobutyric acid (GABA) mediate neuropathic pain comorbid with anxiety through multiple pathways. Immune pathway (Red): gut dysbiosis leads to LPS activating the TLR4-NF-κB pathway, releasing large amounts of inflammatory cytokines into the bloodstream. These cytokines cross the blood-brain barrier (BBB), inducing central inflammation and triggering NP-anxiety comorbidity. Metabolic pathways (Yellow, Green): decreased SCFAs weaken their dual anti-inflammatory and neuroprotective effects, which involve activating FFAR2/3 receptors to release IL-10 and inhibit the NF-κB pathway. Reduced bile acid levels impair their inhibitory effect on the NLRP3 inflammasome via the TGR5/cAMP-PKA signaling axis. Neural pathway (Blue): abnormalities in 5-HT and GABA regulate receptor expression, neuroinflammation, and neuroplasticity in the brain via the vagus nerve, participating in the development of comorbidity. Created with BioGDP.com (Jiang S. et al., 2025).

The human gut coexists with trillions of microorganisms, forming a dynamically balanced system (Adak and Khan, 2019). This microbial community and the host achieve complex bidirectional communication via the “microbiota-gut-brain axis” (Zhuang et al., 2024). This axis is a network integrating neural, endocrine, immune, and metabolic pathways: the neural pathway, centered on the vagus nerve combined with sympathetic and parasympathetic nerve fibers, is responsible for rapidly transmitting chemical and mechanical signals from the gut to the central nervous system and receiving descending regulatory commands from the brain (Wu et al., 2021); the endocrine pathway, represented by the hypothalamic-pituitary-adrenal (HPA) axis, can be modulated by gut microbiota metabolites to regulate the release of stress hormones like cortisol, influencing mood and pain modulation (Rusch et al., 2023); the microbial metabolic pathway serves as the material basis, where the gut microbiota converts dietary components into a vast number of biologically active molecules. These molecules enter the circulatory system directly or act on local nerve endings, becoming key chemical messengers linking the gut and brain (Rooks and Garrett, 2016). Figure 1 summarizes how key gut-derived metabolites mediate the comorbidity of neuropathic pain and anxiety through multiple pathways.

The metabolites produced by the gut microbiota are diverse, primarily including: those regulating immune and barrier functions such as lipopolysaccharide (LPS), short-chain fatty acids (SCFAs), and bile acids (BAs) (Wang et al., 2023); and those serving directly as neurotransmitters such as serotonin (5-HT) and γ-aminobutyric acid (GABA) (Ahmed et al., 2022; Chen et al., 2022). These substances may all influence neural function and immune responses via the gut-brain axis. Studies indicate that metabolites, neurotransmitters, and secondary neuromodulators from the gut microbiota can regulate peripheral and central sensitization by targeting corresponding receptors, participating in chronic pain progression (Liu et al., 2023). Furthermore, the prevalent abnormalities in gut microbiota and metabolite profiles in patients with anxiety and depression suggest their potential as novel targets for mood disorder intervention (Yuan et al., 2021). The mechanism of gut microbiota-derived metabolites regulating NP and anxiety comorbidity is summarized in Table 1.

**TABLE 1:** Regulation of gut microbiota-derived metabolites on comorbidity of NP and anxiety.

Metabolite type	Microbiota species	Core pathway/receptor	Effect on NP-anxiety comorbidity	Effector mechanism	References
LPS	Gram-negative bacteria	LR4/NF-κB pathway	Exacerbates	1. Activates the TLR4/NF-κB pathway, driving peripheral and central neuroinflammation. 2. Enhances glutamatergic excitation, inhibits GABAergic inhibition, leading to central sensitization. 3. Activates the HPA axis, upregulates inflammation in limbic brain regions, inducing anxiety.	Di Vincenzo et al., 2024; Ji et al., 2018; Mayerhofer et al., 2017
SCFAs	Acetate: *Bacteroides* spp., *Bifidobacterium* spp.; Propionate: *Bacteroides* spp., *Prevotella* spp. Butyrate: *Clostridium* cluster	FFAR2/FFAR3	Alleviates	1. Inhibits NF-κB via receptor activation, promotes anti-inflammatory factors (e.g., IL-10), reducing neuroinflammation. 2. Promotes synthesis of neurotransmitters like GABA, 5-HT, and epigenetically regulates expression of factors like BDNF.	Palepu et al., 2024; Riehl et al., 2023; Wei et al., 2023
BAs	DCA, LCA: Clostridia clusters XIVa, IV UDCA: *Clostridium absonum*, *Stenotrophomonas maltophilia*, *Ruminococcus gnavus* and *Collinsella aerofaciens*	FXR, TGR5	Alleviates/exacerbates	1. Protective effect: Activates TGR5, which can inhibit neuroinflammation 2. Damaging effect: Abnormal BA levels can induce liver inflammation; inflammatory cytokines entering the brain exacerbate NP and anxiety.	Eggert et al., 2014; Guzior and Quinn, 2021; Lenin et al., 2023; Chen et al., 2025
5-HT	*Corynebacterium* spp., *Streptococcus* spp., and *Escherichia coli*	5-HT receptors (multiple subtypes)	Alleviates/exacerbates (receptor-dependent)	1. Complex regulation: Microbiota affects brain 5-HT levels by modulating precursor tryptophan metabolism. 2. Receptor specificity: 5-HT1 and 5-HT2 receptors promote pain; 5-HT4 and 5-HT1A receptors produce analgesia.	Alfaro-Rodríguez et al., 2024; Sagalajev et al., 2015; Yano et al., 2015; Bianco et al., 2016
GABA	*Lactobacillus* spp.	GABA_A, GABA_B receptors	Alleviates	1. As a major inhibitory neurotransmitter, alleviates central sensitization. 2. Signals can be transmitted via the vagus nerve, regulating GABA receptor expression in the brain and alleviating anxiety.	Barrett et al., 2012; Bravo et al., 2011

LPS, lipopolysaccharide; SCFAs, short-chain fatty acids; BAs, bile acids; 5-HT, serotonin; GABA, γ-aminobutyric acid; DCA, deoxycholic acid; LCA, lithocholic acid; UDCA, ursodeoxycholic acid; HPA, hypothalamic-pituitary-adrenal.

Current research primarily focuses on these five metabolites: SCFAs, BAs, LPS, 5-HT, and GABA. Others, such as indoles, histamine, and dopamine, as secondary neuromodulators, have potential roles in central nervous system regulation (Riehl et al., 2023; Salminen, 2023), but evidence for their specific mechanisms in NP-anxiety comorbidity remains relatively scarce. Therefore, this review chooses to focus on these five representative, well-studied metabolites with relatively complete evidence chains. It systematically analyzes their physiological and pathological roles in NP-anxiety comorbidity, aiming to provide new perspectives for elucidating the mechanisms of this comorbidity and to lay the foundation for future research extending to more microbial metabolites and the development of targeted intervention strategies.

## 2 Lipopolysaccharide

### 2.1 Mechanism in NP

Lipopolysaccharide (LPS), a key component of the outer membrane of Gram-negative bacteria, is effectively contained when the intestinal barrier is intact. However, when gut dysbiosis leads to abnormal proliferation of Gram-negative bacteria or impaired intestinal barrier function, LPS translocates into systemic circulation, becoming a core immune mediator linking gut disturbances to central nervous system inflammation (Brandsma et al., 2019; Weiss and Hennet, 2017).

As a potent immune stimulant, LPS activates Toll-like receptor 4 (TLR4), widely distributed in the gut, immune cells, and the central nervous system (Ramakrishna et al., 2019). This triggers downstream signaling pathways like NF-κB, driving the massive release of pro-inflammatory cytokines such as TNF-α and IL-1β (Linnerbauer et al., 2020; Woodburn et al., 2021). These peripheral inflammatory mediators can cross the blood-brain barrier (BBB) via the circulatory system, directly activating microglia and astrocytes, thereby inducing sustained neuroinflammation. Activated glial cells further release chemokines like CXCL1 and pro-inflammatory mediators, creating an inflammatory microenvironment. This environment leads to significant changes in synaptic plasticity: enhancing excitatory transmission from glutamatergic neurons while inhibiting the inhibitory regulation by GABAergic interneurons, collectively contributing to central sensitization (Ji et al., 2018).

### 2.2 Mechanism in anxiety

The abnormal activation of the immune system and a persistent low-grade inflammatory state have long been recognized as core pathogenic mechanisms of mood disorders like depression and anxiety (Guo et al., 2023). Within this framework, gut-derived LPS, as a potent immune stimulant, provides a key model for studying how peripheral immune activation affects brain function and behavior. Its action chain likely begins with changes in the gut microenvironment. Gut dysbiosis, especially the abnormal proliferation of Gram-negative bacteria, not only directly increases LPS levels but also disrupts intestinal barrier integrity. This “leaky gut” state allows LPS, other microbial metabolites, and neuroactive substances from the gut to enter systemic circulation (Shu et al., 2023). The LPS-TLR4-NF-κB pathway releases large amounts of pro-inflammatory factors peripherally. These peripherally produced inflammatory mediators can activate the brain’s resident immune cells–microglia and astrocytes–via multiple routes (BBB, vagal afferent fibers), inducing central neuroinflammation (Anand et al., 2022; Lu et al., 2023; Sun et al., 2021). Neuroinflammation further affects key brain regions for mood regulation, including the amygdala, prefrontal cortex, and hippocampus, inducing anxiety (Gholami-Mahtaj et al., 2022; Wang et al., 2018; Zheng et al., 2021).

Notably, this process is not unidirectional. On one hand, gut-derived LPS can enter the bloodstream and influence brain function. On the other hand, states like anxiety and stress activate the core stress-regulating system, the hypothalamic-pituitary-adrenal (HPA) axis, promoting elevated adrenal cortisol levels. High cortisol directly damages the intestinal barrier, increasing its permeability, reducing mucus secretion, and downregulating tight junction protein expression, thereby exacerbating structural and functional damage (de Weerth, 2017). Simultaneously, neural and endocrine changes induced by stress alter gut motility, digestive secretion, and local immune status, further disrupting microbial balance and promoting an increase in Gram-negative bacteria and release of more LPS (Zhang H. et al., 2023; Zhang et al., 2024). This LPS re-enters circulation, triggering chronic systemic low-grade inflammation, which in turn feeds back to the brain, forming a self-reinforcing interference loop within the “brain-gut axis.”

### 2.3 Overlapping pathways and shared signaling nodes

A key common pathological basis for the comorbidity of chronic pain and mood disorders is central inflammation (Candelli et al., 2021; Mohammad and Thiemermann, 2020). Clinical studies confirm that LPS can activate systemic immunity, elevate TNF-α, IL-6, and IL-8 levels, thereby exacerbating pain sensitivity and anxiety symptoms (Wegner et al., 2014). Mechanistically, the frequent co-occurrence of pain and anxiety stems from LPS triggering a shared pathological process via the gut-brain axis: it directly activates the LPS/TLR4/NF-κB pathway, causing systemic and central immune responses that directly worsen pain perception; it simultaneously disrupts HPA axis function, leading to persistently abnormal cortisol secretion. The high-cortisol environment induces neuroinflammation in the hippocampus and amygdala and inhibits the action of protective factors like IL-10, synchronously generating anxiety-like behaviors. Therefore, pain and anxiety symptoms induced by LPS are not simply sequential cause and effect but parallel manifestations of the same immune-endocrine disruption across sensory and emotional dimensions (Mahdirejei et al., 2023).

However, the latest evidence does not unidirectionally support the pro-inflammatory role of LPS. LPS from *Rhodobacter sphaeroides* is a highly specific TLR4 antagonist and can regulate nociception-related factors in the dorsal root ganglion, thereby exerting analgesic effects (Jurga et al., 2018). This indicates that the effect of LPS is not absolutely harmful; its impact may depend on factors like its source, target sites, and microenvironment. Future research needs more in-depth critical discussion on such paradoxical findings, e.g., systematically comparing the structure-function relationships of LPS from different sources, clarifying its bidirectional regulatory role at physiological versus pathological concentrations, and exploring its differential effects at various nodes of the gut-brain axis (e.g., gut, peripheral nerves, spinal cord, brain). This comprehensive, dialectical perspective is crucial for accurately understanding the complex role of LPS in NP-anxiety comorbidity.

## 3 Short-chain fatty acids

### 3.1 Mechanism in NP

Short-chain fatty acids (SCFAs) are a class of microbial metabolites primarily composed of acetate, propionate, and butyrate. They are produced in the gut by specific microbial fermentation of dietary fiber: acetate is mainly produced by *Bacteroides* spp. and *Bifidobacterium* spp. (Tsukuda et al., 2021); propionate is predominantly produced by *Bacteroides* spp. and *Prevotella* spp. (Killingsworth et al., 2020); butyrate is mainly produced by Firmicutes, specifically clostridial clusters (Singh et al., 2022). These three SCFAs activate shared or unique free fatty acid receptors (FFARs), such as FFAR2 (GPR43) and FFAR3 (GPR41), collectively maintaining gut homeostasis, enhancing the intestinal barrier, and regulating systemic and neural immunity (Fusco et al., 2023; Mishra et al., 2020).

Short-chain fatty acids levels are closely related to NP. A study in the NP rat models revealed an increased Firmicutes/Bacteroidetes ratio accompanied by significantly reduced acetate and butyrate levels. *Lactobacillus plantarum* supplementation can exert analgesic effects by restoring SCFA levels, promoting anti-inflammatory IL-10 secretion, and inducing macrophage polarization from pro-inflammatory M1 to anti-inflammatory M2 phenotype (Huang et al., 2024). Therefore, SCFAs are not only potential biomarkers for NP, but the anti-inflammatory and immunomodulatory mechanisms they mediate are also core targets for intervening in NP and related comorbidities.

New research finds different SCFAs have varying effects on immune and neural cells:

Acetate is a key microbe-derived molecule driving microglial maturation and functional regulation. It can cross the blood-brain barrier (Fock and Parnova, 2023) and regulate microglial homeostasis and function by modulating histone modifications, influencing the progression of neuroinflammation-related diseases (Erny et al., 2021; Lynch et al., 2021).

The neuroprotective effect of propionate primarily relies on its targeting of FFAR3 and regulation of the epigenetic state. Under neuroinflammatory conditions, propionate specifically activates FFAR3, thereby inducing hyperacetylation of histone H3. This epigenetic remodeling not only directly enhances the resistance of neurons and glial cells to oxidative stress but also promotes the expression of neuroregeneration markers like growth-associated protein-43 by regulating related gene expression, supporting neural repair (Grüter et al., 2023).

Butyrate is the primary energy source for colonocytes and is crucial for maintaining intestinal barrier integrity (Geirnaert et al., 2017). Systemically, it exerts immunomodulatory effects by activating the aryl hydrocarbon receptor (AhR) and G protein-coupled receptors (e.g., GPR109a, GPR43) (Singh et al., 2014). These signaling pathways promote the production of the anti-inflammatory factor IL-10, enhance regulatory T cell function, and inhibit Th1/Th17 cell differentiation and the release of pro-inflammatory mediators like IL-17, thereby alleviating systemic low-grade inflammation (Kasubuchi et al., 2015). Within the central nervous system, butyrate directly inhibits excessive microglial activation by activating the GPR109A receptor, upregulating PPAR-γ, and inhibiting the TLR4/NF-κB signaling pathway. It corrects M1/M2 polarization imbalance, reduces pro-inflammatory factor release, demonstrating direct neuroprotective properties (Wang et al., 2022; Wei et al., 2023).

In summary, SCFAs, through their microbiota-specific production, multi-receptor-mediated signaling, and epigenetic regulation, constitute an integrative protective network from gut to brain. In NP states, abnormal or insufficient SCFA levels may weaken this endogenous anti-inflammatory and neuroprotective barrier.

### 3.2 Mechanism in anxiety

Short-chain fatty acids produced by gut microbiota metabolism possess the ability to modulate brain cognition and behavior (Mirzaei et al., 2021). Clinical studies show that the abundance of SCFA-producing bacteria is significantly reduced in the guts of anxiety patients, and the severity of anxiety disorder symptoms is positively correlated with Firmicutes abundance (Jiang et al., 2018; Ketel et al., 2024). This is because FFAR2 and FFAR3 are expressed not only in the enteric nervous system (ENS) but also in the portal vein nerves and sensory ganglia, forming the structural basis for gut-brain signal transmission (Dicks, 2022). Among these, FFAR3 in the ENS can directly transmit signals generated by SCFAs to the central nervous system (CNS), thereby influencing brain function and behavior (Nøhr et al., 2013). Blocking this pathway induces gut leakiness, neuroinflammation, and anxiety-like behaviors, while butyrate supplementation effectively alleviates these abnormalities (Mishra et al., 2024). Mechanistically, SCFAs not only inhibit systemic and neuroinflammatory responses by regulating microglial activity (Fusco et al., 2023), but also promote the synthesis of neurotransmitters like 5-HT and GABA, directly modulating mood circuits (Nankova et al., 2014; Palepu et al., 2024). This indicates that SCFAs maintain emotional homeostasis by mediating gut-brain dialogue and exerting anti-inflammatory and neuromodulatory functions; their deficiency or impaired signaling is an important mechanism in the development of anxiety.

### 3.3 Overlapping pathways and shared signaling nodes

Gut dysbiosis not only leads to the accumulation of harmful metabolites (e.g., LPS) but also directly causes reduced synthesis of beneficial metabolites like SCFAs. This metabolic imbalance constitutes an important pathological basis for NP-anxiety comorbidity (Park and Kim, 2021). As key messengers of the gut-brain axis, SCFAs exert neuroprotective effects through the following multiple mechanisms: first, enhancing the structural and functional integrity of both the intestinal barrier and the blood-brain barrier (Herrera et al., 2024); second, regulating gene transcription by inhibiting histone deacetylase (HDAC) activity, and specifically activating FFAR2/FFAR3, thereby inhibiting classical inflammatory pathways like NF-κB, exerting potent anti-inflammatory effects (Kopczyñska and Kowalczyk, 2024; Zhang K. et al., 2023). These mechanisms collectively regulate systemic and neural immune homeostasis, effectively alleviating neuroinflammation-driven pain sensitization and anxiety-like behaviors.

More importantly, SCFAs can act as epigenetic regulators, directly or indirectly modulating the expression of key neurotrophic factors like brain-derived neurotrophic factor (BDNF) in the brain via the “microbiota-gut-brain axis” (Dalile et al., 2019). This may be a core mechanism through which they improve neuroplasticity and intervene in comorbidity. BDNF itself is a key molecule in pain-emotion comorbidity: it activates microglia via purinergic receptors P2×4R and P2×7R, promoting neuroinflammation and other mechanisms that trigger central sensitization, forming the basis of pain (Hu et al., 2022; Rajamanickam et al., 2024). This signal further ascends, participating in the development of anxiety comorbidity by modulating excitability in emotional brain regions like the thalamus, cortex, and limbic system (Pezet et al., 2002; Smith, 2024).

In NP-anxiety comorbidity, SCFAs act as both protectors of the gut and blood-brain barriers and as upstream core regulators. The deficiency of SCFAs not only weakens their direct neuroprotective effects but may also promote the occurrence and development of NP-anxiety comorbidity at multiple levels by disrupting the normal regulation of pathways like BDNF. However, it remains unclear how SCFAs precisely regulate BDNF and other signaling molecules to mediate gut-brain dialogue and influence NP-anxiety comorbidity progression, and whether they can become potential intervention targets for this comorbidity. Future research is needed to clarify these points.

## 4 Bile acids

### 4.1 Mechanism in NP

Approximately 90% of bile acids (BAs) are synthesized from cholesterol in the liver and subsequently transported to the intestine, where they are metabolized by gut microbiota (Chávez-Talavera et al., 2017). The biological functions of BAs are primarily realized through binding to the farnesoid X receptor (FXR) and the G protein-coupled bile acid receptor 5 (TGR5) (Jiang X. et al., 2025). Receptor activation can enhance the inhibitory function of GABAergic neurons, thereby inhibiting abnormal activation of glial cells like microglia in the spinal dorsal horn, downregulating activation of their downstream ERK signaling pathway, and ultimately reducing neuronal sensitization and pain (Wu et al., 2023).

However, the opposite occurs in systemic metabolic disease states like diabetes. Gut dysbiosis-induced bile acid metabolism abnormalities lead to abnormal activation of the TGR5 - cyclic adenosine monophosphate (cAMP) - cAMP response element-binding protein (CREB) signaling pathway (Chen et al., 2024). Activated CREB binds to the promoter region of the transient receptor potential vanilloid type 1 (TRPV1) channel gene, directly upregulating TRPV1 transcription and protein expression (Cenac et al., 2015). The TRPV1 protein is enriched at nerve terminals (Tominaga et al., 1998); when stimulated by heat, protons, or endogenous lipid mediators, the channel opens more readily, causing massive calcium influx (Sanz-Salvador et al., 2012), triggering neuronal depolarization and high-frequency action potential firing, i.e., peripheral sensitization (Bujak et al., 2019; Clapham, 2003). TRPV1 activation further activates protein kinase C (PKC), which can directly modulate the function of TRPV1 and other ion channels (e.g., sodium channels) through phosphorylation, forming a positive feedback loop that continuously amplifies pain signals (Gao et al., 2024). This pathway not only provides a new mechanistic explanation for diseases like diabetic peripheral neuropathic pain but also suggests that targeting TGR5 or interrupting its downstream signaling may become a potential therapeutic strategy for alleviating such NP.

### 4.2 Mechanism in anxiety

Bile acids synthesized by hepatocytes are metabolized by gut microbiota into secondary BAs, including deoxycholic acid (DCA), lithocholic acid (LCA), and ursodeoxycholic acid (UDCA) (Chand et al., 2017). Studies have found that abnormal elevation of specific bile acids and their derivatives is associated with anxiety (Wang et al., 2024). For example, gut dysbiosis can lead to increased serum LCA levels, inducing liver inflammation. The resulting inflammatory cytokines may cross the blood-brain barrier, exacerbating neuroinflammation in the brain and leading to anxiety-like behaviors (Weng et al., 2024). Furthermore, bile acids can enter the CNS via the circulatory system. TGR5 is widely expressed in the CNS, enabling BAs to directly regulate neural function (Joyce and O’Malley, 2022). Taurodeoxycholic acid can directly activate central TGR5 receptors, exerting protective effects by inhibiting neuroinflammation, oxidative stress, and endoplasmic reticulum stress (Lenin et al., 2023). Conversely, TGR5 gene deletion leads to decreased serum and hippocampal serotonin (5-HT) levels in mice and induces anxiety- and depression-like behaviors (Castellanos-Jankiewicz et al., 2021; Tao et al., 2024). Therefore, TGR5 is both a key molecule mediating neuroprotection, and its functional loss is a direct cause of mood disorders. Its ultimate effect likely depends highly on factors such as the specificity of the activating ligand and the cellular and brain regional microenvironment (Chen et al., 2023). Thus, simply defining it as a “protective pathway” may not be comprehensive. Although existing evidence demonstrates the critical role of the BA-TGR5 pathway in anxiety regulation, its downstream molecular mechanisms require further study.

### 4.3 Overlapping pathways and shared signaling nodes

The comorbidity of NP and anxiety stems from the synergistic effects of abnormal primary bile acid synthesis and signaling pathway dysregulation (Ridlon and Bajaj, 2015; Zhang J. et al., 2023). In pathological states, excessive specific bile acids or receptor function imbalance can simultaneously activate glial cells in the spinal dorsal horn, hippocampus, and amygdala, releasing pro-inflammatory factors and creating widespread central neuroinflammation (Chen et al., 2025; Shi et al., 2025). Delving into the molecular mechanism, bile acids can induce phosphorylation and ubiquitination of the NLRP3 inflammasome by activating the TGR5-cAMP-protein kinase A (PKA) axis, thereby directly inhibiting the activation of this key inflammatory complex (Guo et al., 2016). Therefore, bile acid signaling dysregulation weakens the physiological inhibition of the NLRP3 inflammasome, consequently synchronously exacerbating neuroinflammatory responses in both the spinal cord and limbic system; conversely, restoring signaling balance can curb this shared pathway from the upstream. This inflammatory process not only directly lowers the pain threshold, causing hyperalgesia/allodynia but also simultaneously impairs hippocampal neuroplasticity, disrupts amygdala fear/anxiety-related neural circuits, and affects the balance of key neurotransmitter systems like 5-HT and GABA (Huang et al., 2015; Romanazzi et al., 2023). Additionally, the bile acid-TGR5-cAMP-CREB-TRPV1 pathway may also be co-activated in this process, exacerbating neuroinflammation and further participating in the regulation of molecular events related to pain and emotion. In summary, the bile acid-TGR5-cAMP signaling pathway is a key shared signaling node in NP-anxiety comorbidity. Activation of this pathway can directly regulate neuronal excitability in brain regions integrating pain and emotion. More critically, this signal, by acting on different downstream molecules, triggers a shared and diffusible neuroinflammatory pathway, thereby contributing to the comorbid state of NP and anxiety (Zhong et al., 2023).

## 5 Neurotransmitters: 5-HT and GABA

### 5.1 Mechanism in NP

Serotonin (5-HT) is an important gastrointestinal signaling molecule with distinct synthetic sources and functional divisions in the central and peripheral systems: in the CNS, 5-HT is primarily synthesized by raphe nucleus neurons in the brainstem, involved in mood, sleep, and anxiety regulation (Jones et al., 2020); in the periphery, 90%–95% of 5-HT is synthesized by enterochromaffin cells (Gershon and Tack, 2007), its release directly regulated by gut microbiota like lactobacilli (Ling et al., 2022; Lu et al., 2021), and it mainly participates in gastrointestinal function regulation (Bulbring and Lin, 1958).

The vagus nerve is a core pathway for gut-brain communication, originating from the medulla oblongata and widely distributed in organs like the gastrointestinal tract (Dicks, 2023). Vagus nerve activation can significantly alter neurotransmitter levels, thereby affecting digestion, immunity, and gut microbiota composition and metabolism, forming the basis of neural regulation (Shin et al., 2019). Under pathological conditions, gut-derived 5-HT activates 5-HT3 and 5-HT4 receptors on vagal afferent fibers, uploading nociceptive information to the CNS (Brito et al., 2017; Nemoto et al., 2001). Simultaneously, 5-HT also participates in the formation of hyperalgesia by acting on 5-HT1 and 5-HT2 receptors in the spinal cord (Alfaro-Rodríguez et al., 2024; Sagalajev et al., 2015), while activation of 5-HT4 and 5-HT1A receptors in the ENS exhibits analgesic and neuroprotective properties (Bianco et al., 2016). Gut dysbiosis may disrupt this balance, leading to enhanced abnormal vagal afferent input and weakened spinal analgesic function, exacerbating NP. This indicates the functional duality of 5-HT in NP (Liu et al., 2020). Notably, these receptors likely act synergistically; differences in NP etiology and dynamic regulation of 5-HT levels by gut microbiota metabolites may influence the predominant expression and function of different 5-HT receptor subtypes (Zhang et al., 2025), and detailed mechanisms require further investigation.

γ-aminobutyric acid is an inhibitory neurotransmitter that also plays an important role in maintaining ENS structure and functional regulation, participating in the control of gastric acid secretion, gastric emptying, intestinal motility, and pain perception, thus possessing dual functions as both a neurotransmitter and an endocrine modulator (Chen et al., 2022). Studies show that gut microbiota such as *Lactobacillus*, *Bifidobacterium*, *Bacteroides*, and *B. fragilis* have the ability to synthesize GABA (Strandwitz et al., 2019). GABA and its receptors are widely distributed in the ENS (Auteri et al., 2015), involved in regulating the sensitivity of vagal and spinal afferent nerves (Hyland and Cryan, 2010). Vagus nerve activation can transmit gut signals to the brainstem, subsequently activating GABAergic descending inhibitory pathways in the brain. This pathway further synergizes with the 5-HT system, collectively enhancing inhibition on pain-transmitting neurons in the spinal cord or trigeminal spinal nucleus, thereby achieving pain modulation (Cornelison et al., 2020).

Although gut-derived GABA itself has difficulty crossing the BBB (Janeczko et al., 2007), a small amount may enter the CNS via specific transporters on the BBB (Takanaga et al., 2001), regulating uptake processes at nerve terminals and glial cells (Brown et al., 2003). Furthermore, gut microbiota may achieve cross-system regulation through other means. For example, *Akkermansia muciniphila*, *Parabacteroides merdae*, and *Parabacteroides distasonis* can indirectly influence GABAergic signaling and thus pain perception by modulating peripheral GABA/glutamate metabolic balance (Olson et al., 2018; Pokusaeva et al., 2017). This suggests that the gut microbiota-GABA signal may achieve central regulation through direct transport or other indirect pathways; its specific molecular mechanisms require in-depth elucidation (Boonstra et al., 2015).

### 5.2 Mechanism in anxiety

Tryptophan (TRP) and its key metabolite 5-HT are central to anxiety regulation. In 5-HT biosynthesis, TRP is first catalyzed by tryptophan hydroxylase (TPH) into 5-hydroxytryptophan, then converted to 5-HT via aromatic L-amino acid decarboxylase. TPH1 is mainly present peripherally (e.g., in enterochromaffin cells), while TPH2 is present in central neurons (Qu et al., 2024). Gut microbiota can control the shunt of TRP metabolism toward the kynurenine versus 5-HT synthesis pathways, promoting the entry of BBB-permeable tryptophan and 5-hydroxytryptophan into the CNS, thereby indirectly regulating brain 5-HT levels (Clarke et al., 2013; Deng et al., 2021). Fluctuations in 5-HT levels, particularly in the amygdala, hippocampus and striatum, are closely related to anxiety symptoms (Šimić et al., 2021). Disruption of this regulatory network may be an important mechanism causing anxiety. Studies show that *Toxoplasma gondii* infection can ultimately lead to reduced 5-HT levels and neuroinflammation in key brain regions like the amygdala by disrupting gut microbiota, thereby inducing anxiety-like behaviors (Luo et al., 2024). Conversely, probiotic intervention demonstrates the potential to improve mood by positively modulating this pathway. For instance, *Lactobacillus paracasei* PS23 can elevate 5-HT levels in the hippocampus and striatum and effectively alleviate anxiety behaviors (Liao et al., 2019).

Dysfunction of the GABAergic system has been confirmed to be closely associated with the occurrence of anxiety. Gut microbiota (e.g., lactobacilli, bifidobacteria) possess the ability to synthesize GABA (Włodarczyk et al., 2020) or influence GABA synthesis by regulating levels of its key precursor, glutamine (Zhu et al., 2024). Additionally, gut microbiota can stimulate vagal afferents, transmitting peripheral signals to the brainstem, subsequently regulating GABA receptor expression and function in the limbic system, enhancing central inhibitory neurotransmission, thereby alleviating anxiety (Bravo et al., 2011). Conversely, in pathological states, gut dysbiosis may lead to abnormal elevation of short-chain fatty acids (especially butyrate), which can interfere with normal inhibitory signal transmission by competitively binding to GABA receptors, inducing anxiety-like behaviors (Dicks, 2023).

This indicates that gut dysbiosis can promote anxiety by reducing GABA synthesis, producing receptor-antagonist metabolites, and weakening vagal transmission, leading to central GABAergic inhibitory function deficits; however, supplementing specific probiotics can reverse these pathway alterations, restoring inhibitory function and synaptic efficacy, ultimately alleviating anxiety-like behaviors (Isik et al., 2025; Ma et al., 2021).

### 5.3 Overlapping pathways and shared signaling nodes

Increasing evidence suggests that dysfunctional interaction between vagal signaling and gut microbiota-derived mediators is a key link connecting chronic pain and mood disorders (Kurhaluk et al., 2025). Its core mechanism begins with gut dysbiosis and the resulting microenvironmental changes, relying on the vagus nerve to transmit signals to the brain. Ultimately, they collectively regulate the development of pain and anxiety by modulating the expression of specific brain receptors, influencing neuroinflammation and neuroplasticity. Severing this nerve blocks the central regulatory effects of both 5-HT and GABA (Lee et al., 2025; Zou et al., 2024). However, their modes of action differ. 5-HT is primarily released locally in the gut and initiates signal upload to the nucleus tractus solitarius via direct activation of 5-HT receptors on vagal nerve endings, with projections to brain regions like the prefrontal cortex, hippocampus, and amygdala–a relatively direct pathway (Lee et al., 2025). The role of the vagus nerve in GABAergic gut-brain communication is more indirect and complex: it may influence signal transmission by modulating the local gut environment or vagal nerve function, and its effects are significantly context-dependent. Studies find that in pathological states like *Salmonella* infection, the vagus nerve may continuously transmit pro-anxiety signals. Probiotics can induce changes in GABA and its receptor levels in the blood and brain limbic system via the vagus nerve, alleviating pain and anxiety (Bravo et al., 2011; Royo et al., 2023). This indicates that the vagus nerve is not merely a simple signal conduit but an active, functionally selective pathway (Faraji et al., 2025). It is responsible for precisely uploading signals from gut microbiota-derived metabolites and induced immune/neuroendocrine signals to the brainstem, subsequently influencing neurotransmitter receptor expression and functional remodeling in brain regions like the limbic system, ultimately achieving regulation of NP and anxiety (Cryan et al., 2019; Yu and Hsiao, 2021).

## 6 Other gut microbiota-derived metabolites

Besides the five metabolites mentioned above, research has found that the fecal microbiota of irritable bowel syndrome patients is enriched with histamine-producing bacteria. Their metabolically produced histamine can activate local gut and systemic histamine receptors, promoting mast cell activation and inducing visceral hyperalgesia (De Palma et al., 2022). Another study found that indoles, metabolites produced by gut microbiota, can serve as key signaling molecules, participating in anxiety regulation by modulating amygdala neuroplasticity (Yu et al., 2025). The anxiolytic effect of *Lactobacillus plantarum* PS128 has been found to be associated with increased striatal dopamine levels (Liu et al., 2016). These studies suggest that gut-derived secondary neurotransmitter modulators like indoles, histamine, and dopamine may regulate pain-anxiety comorbidity through different mechanisms. However, this field currently lacks direct evidence, and their specific molecular mechanisms are not fully elucidated. Future research is needed to clarify their physiological and pathological significance.

## 7 Potential therapeutic strategies

The comorbidity of chronic pain and anxiety, while clinically involving separate abnormalities in sensory and emotional systems, may share an upstream origin in the dysregulation of gut microbiota metabolic networks. Therefore, intervening in the gut microbial community and its metabolic profile to restore homeostasis holds promise as a potential novel strategy for simultaneously alleviating both pain and anxiety. Currently, various intervention methods (e.g., probiotics, dietary intervention, microbiota transplantation) have shown positive effects in treating pain and anxiety. Specific research examples and their mechanisms of action are summarized in Table 2.

**TABLE 2:** Clinical and preclinical studies on targeted intervention of gut microbiota for pain and anxiety.

Intervention type	Intervention	Model/subject	Key metabolite/ molecule	Pain/anxiety effect	Mechanism	References
Probiotics	Multiple probiotics	PDN rats	LPS, TLR4/MyD88/NF-κB pathway	Alleviates pain	Repairs intestinal barrier, reduces serum LPS and pro-inflammatory factors, inhibits TLR4/MyD88/NF-κB pathway	Jiang S. et al., 2025
	*Lactobacillus reuteri* DSM 17938	LPS-induced depression/anxiety mice	Amino acid, unsaturated fatty acid	Alleviates depression and anxiety-like behavior	Restores gut microbiota composition, corrects metabolic pathway disturbances in hippocampus and prefrontal cortex	Mo et al., 2025
	Psychobiotic (mainly *Lactobacillus reuteri*)	BPA-induced NP with anxiety mice	Bile acids, neurotransmitter amino acid metabolites	Alleviates anxiety-like behavior	Modulates bile acid metabolism and neurotransmitter amino acids, improves gut microbiota structure	Zhang H. et al., 2023
Prebiotics	Inulin	Alcohol withdrawal mice	SCFAs, 5-HT, TPH1	Alleviates anxiety and depression-like behavior	Increases abundance of SCFA-producing bacteria, elevates 5-HT precursor 5-HTP, modulates tryptophan metabolism	Li et al., 2024
Postbiotics	Postbiotic Probio-Eco^®^	Chronic diarrhea patients	Butyrate, tryptophan metabolites, bile acids	Alleviates anxiety	Increases beneficial bacteria, elevates butyrate, modulates tryptophan-5-HT and bile acid metabolism	Guo et al., 2024
Dietary intervention	Ketogenic diet	Healthy C57BL/6J mice	BHB, IL-1β, IL-10	Alleviates anxiety in males	Increases BHB levels, inhibits microglial activation, elevates anti-inflammatory factor IL-10	Kumar et al., 2024
	Hydrogen-rich water	CINP mice	LPS, TLR4, TNF-α, IL-6	Alleviates pain	Modulates gut microbiota structure, reduces LPS-TLR4 pathway activity, decreases oxidative stress and inflammation	Lian et al., 2021
Metabolite supplementation	SCFA supplements (acetate, propionate, butyrate)	TRD rats	SCFAs, 5-HT, GABA, norepinephrine, dopamine | Antidepressant and anxiolytic effects	Alleviates anxiety and depression-like behavior	Restores neurotransmitter levels, reduces inflammation, strengthens gut barrier, modulates tryptophan metabolism	Palepu et al., 2024
Microbial transplantation	Fecal microbiota transplantation	IBD patients (with bile acid malabsorption)	Bile acid synthesis-related metabolites	Alleviates diarrhea and abdominal pain	Modulates bile acid metabolism, restores microbiota structure, reduces inflammatory markers	Lu et al., 2025

LPS, lipopolysaccharide; SCFAs, short-chain fatty acids; 5-HT, serotonin; 5-HIAA, 5-hydroxyindoleacetic acid; 5-HTP, 5-hydroxytryptophan; GABA, γ-aminobutyric acid; BHB, β-hydroxybutyrate; TPH1, tryptophan hydroxylase 1;PDN, painful diabetic neuropathy; BPA, brachial plexus avulsion; CINP, chemotherapy-induced neuropathic pain; TRD, treatment-resistant depression; IBD, inflammatory bowel disease.

## 8 Conclusion and future directions

The gut microbiota, as a vast and complex “microbial organ” in the human body, exerts influences far beyond the digestive system, profoundly shaping the host’s physiological and pathological states. Its core role lies in constituting a dynamic systemic regulatory network through multiple parallel and intertwined pathways: metabolism, immunity, neuroendocrinology, and barrier protection. By decomposing dietary components, the gut microbiota produces thousands of metabolites. These molecules are not only regulators of the local environment but also key systemic messengers, modulating the functions of distant organs via the gut-brain axis and other routes. Among the numerous metabolites, LPS, as a potent immune trigger, disrupts the gut barrier, and induce systemic and neurological inflammation by activating the TLR4/NF-κB pathway. In contrast, SCFAs, based on maintaining intestinal barrier integrity and energy supply, exert anti-inflammatory and neuroprotective effects by activating FFAR2/FFAR3, helping maintain immune and neural homeostasis. BAs, synthesized in the liver and metabolically transformed by gut microbiota, play important roles in regulating peripheral and central neuroinflammation and influencing GABAergic neuronal activity by activating receptors like FXR and TGR5. Furthermore, the gut microbiota produces or regulates various neuroactive substances, such as 5-HT precursors, GABA, indoles, histamine, and dopamine. These substances can directly influence enteric and central nervous system functions, participating in pain perception, mood, and behavioral regulation.

However, most current evidence remains at the correlational level, lacking in-depth validation of causal mechanisms. For example, the dynamic transport processes of specific metabolites within the gut-brain axis, their specific cellular targets in the CNS, and their signaling network have not been systematically elucidated. Furthermore, the synergistic or antagonistic effects between different metabolites, as well as their temporal changes during different stages of pain, require further study. Finally, translational bridging between existing animal models and clinical samples is still insufficient, and individual differences and gender specificity in gut microbiota metabolite regulation are often overlooked. Future research needs to integrate multi-level research approaches to deeply dissect the causal roles of key metabolites mechanistically. It should also advance large-cohort clinical studies to develop personalized intervention strategies targeting gut microbiota metabolites, with the aim of providing new avenues for the prevention and treatment of neuropathic pain and anxiety comorbidity.

## References

[B1] Adak A. Khan M. (2019). An insight into gut microbiota and its functionalities. *Cell. Mol. Life Sci*. 76 473–493. 10.1007/s00018-018-2943-4 30317530 PMC11105460

[B2] Ahmed H. Leyrolle Q. Koistinen V. Kärkkäinen O. Layé S. Delzenne N. (2022). Microbiota-derived metabolites as drivers of gut-brain communication. *Gut Microbes* 14 2102878. 10.1080/19490976.2022.2102878 35903003 PMC9341364

[B3] Alfaro-Rodríguez A. Reyes-Long S. Roldan-Valadez E. González-Torres M. Bonilla-Jaime H. Bandala C. (2024). Association of the serotonin and kynurenine pathways as possible therapeutic targets to modulate pain in patients with fibromyalgia. *Pharmaceuticals* 17:1205. 10.3390/ph17091205 39338367 PMC11434812

[B4] Anand N. Gorantla V. Chidambaram S. (2022). The role of gut dysbiosis in the pathophysiology of neuropsychiatric disorders. *Cells* 12:54. 10.3390/cells12010054 36611848 PMC9818777

[B5] Attal N. Bouhassira D. Colvin L. (2023). Advances and challenges in neuropathic pain: A narrative review and future directions. *Br. J. Anaesth*. 131 79–92. 10.1016/j.bja.2023.04.021 37210279

[B6] Auteri M. Zizzo M. Serio R. (2015). GABA and GABA receptors in the gastrointestinal tract: From motility to inflammation. *Pharmacol. Res.* 93 11–21. 10.1016/j.phrs.2014.12.001 25526825

[B7] Barrett E. Ross R. O’Toole P. Fitzgerald G. Stanton C. (2012). γ-Aminobutyric acid production by culturable bacteria from the human intestine. *J. Appl. Microbiol*. 113 411–417. 10.1111/j.1365-2672.2012.05344.x 22612585

[B8] Battaglia M. Garon-Carrier G. Brendgen M. Feng B. Dionne G. Vitaro F. (2020). Trajectories of pain and anxiety in a longitudinal cohort of adolescent twins. *Depress Anxiety* 37 475–484. 10.1002/da.22992 31944483

[B9] Bianco F. Bonora E. Natarajan D. Vargiolu M. Thapar N. Torresan F. (2016). Prucalopride exerts neuroprotection in human enteric neurons. *Am. J. Physiol. Gastrointest Liver Physiol*. 310 G768–G775. 10.1152/ajpgi.00036.2016 26893157 PMC5243219

[B10] Boonstra E. de Kleijn R. Colzato L. Alkemade A. Forstmann B. Nieuwenhuis S. (2015). Neurotransmitters as food supplements: The effects of GABA on brain and behavior. *Front. Psychol.* 6:1520. 10.3389/fpsyg.2015.01520 26500584 PMC4594160

[B11] Brandsma E. Kloosterhuis N. Koster M. Dekker D. Gijbels M. van der Velden S. (2019). A proinflammatory gut microbiota increases systemic inflammation and accelerates atherosclerosis. *Circ. Res*. 124 94–100. 10.1161/CIRCRESAHA.118.313234 30582442 PMC6325767

[B12] Bravo J. Forsythe P. Chew M. Escaravage E. Savignac H. Dinan T. (2011). Ingestion of Lactobacillus strain regulates emotional behavior and central GABA receptor expression in a mouse via the vagus nerve. *Proc. Natl. Acad. Sci. U S A*. 108 16050–16055. 10.1073/pnas.1102999108 21876150 PMC3179073

[B13] Brito R. Rasmussen L. Sluka K. (2017). Regular physical activity prevents development of chronic muscle pain through modulation of supraspinal opioid and serotonergic mechanisms. *Pain Rep.* 2:e618. 10.1097/PR9.0000000000000618 29392233 PMC5777681

[B14] Brown A. Goldsworthy S. Barnes A. Eilert M. Tcheang L. Daniels D. (2003). The Orphan G protein-coupled receptors GPR41 and GPR43 are activated by propionate and other short chain carboxylic acids. *J. Biol. Chem.* 278 11312–11319. 10.1074/jbc.M211609200 12496283

[B15] Bujak J. Kosmala D. Szopa I. Majchrzak K. Bednarczyk P. (2019). Inflammation, cancer and immunity-implication of TRPV1 channel. *Front. Oncol*. 9:1087. 10.3389/fonc.2019.01087 31681615 PMC6805766

[B16] Bulbring E. Lin R. (1958). The effect of intraluminal application of 5-hydroxytryptamine and 5-hydroxytryptophan on peristalsis; the local production of 5-HT and its release in relation to intraluminal pressure and propulsive activity. *J. Physiol.* 140 381–407. 13514713 PMC1358765

[B17] Candelli M. Franza L. Pignataro G. Ojetti V. Covino M. Piccioni A. (2021). Interaction between lipopolysaccharide and gut microbiota in inflammatory bowel diseases. *Int. J. Mol. Sci.* 22:6242. 10.3390/ijms22126242 34200555 PMC8226948

[B18] Castellanos-Jankiewicz A. Guzmán-Quevedo O. Fénelon V. Zizzari P. Quarta C. Bellocchio L. (2021). Hypothalamic bile acid-TGR5 signaling protects from obesity. *Cell. Metab.* 33 1483–1492.e10. 10.1016/j.cmet.2021.04.009 33887197

[B19] Cenac N. Bautzova T. Le Faouder P. Veldhuis N. Poole D. Rolland C. (2015). Quantification and potential functions of endogenous agonists of transient receptor potential channels in patients with irritable bowel syndrome. *Gastroenterology* 149 433–444.e7. 10.1053/j.gastro.2015.04.011 25911511

[B20] Chand D. Avinash V. Yadav Y. Pundle A. Suresh C. Ramasamy S. (2017). Molecular features of bile salt hydrolases and relevance in human health. *Biochim. Biophys. Acta Gen. Subj.* 1861 2981–2991. 10.1016/j.bbagen.2016.09.024 27681686

[B21] Chávez-Talavera O. Tailleux A. Lefebvre P. Staels B. (2017). Bile acid control of metabolism and inflammation in obesity, type 2 diabetes, dyslipidemia, and nonalcoholic fatty liver disease. *Gastroenterology* 152 1679–1694.e3. 10.1053/j.gastro.2017.01.055 28214524

[B22] Chen M. Ruan G. Chen L. Ying S. Li G. Xu F. (2022). Neurotransmitter and intestinal interactions: Focus on the microbiota-gut-brain axis in irritable bowel syndrome. *Front. Endocrinol*. 13:817100. 10.3389/fendo.2022.817100 35250873 PMC8888441

[B23] Chen P. Jiang X. Fu J. Ou C. Li Y. Jia J. (2024). The potential mechanism of action of gut flora and bile acids through the TGR5/TRPV1 signaling pathway in diabetic peripheral neuropathic pain. *Front. Endocrinol.* 15:1419160. 10.3389/fendo.2024.1419160 39619328 PMC11604420

[B24] Chen S. Shao Q. Chen J. Lv X. Ji J. Liu Y. (2023). Bile acid signalling and its role in anxiety disorders. *Front. Endocrinol.* 14:1268865. 10.3389/fendo.2023.1268865 38075046 PMC10710157

[B25] Chen X. Zhou Q. He Y. Wang Y. Jiang Y. Ren Y. (2025). TGR5 dysfunction underlies chronic social defeat stress via cAMP/PKA signaling pathway in the hippocampus. *Transl. Psychiatry* 15:366. 10.1038/s41398-025-03599-7 41053031 PMC12501386

[B26] Clapham D. E. (2003). TRP channels as cellular sensors. *Nature* 426 517–524. 10.1038/nature02196 14654832

[B27] Clarke G. Grenham S. Scully P. Fitzgerald P. Moloney R. Shanahan F. (2013). The microbiome-gut-brain axis during early life regulates the hippocampal serotonergic system in a sex-dependent manner. *Mol. Psychiatry* 18 666–673. 10.1038/mp.2012.77 22688187

[B28] Colloca L. Ludman T. Bouhassira D. Baron R. Dickenson A. Yarnitsky D. (2017). Neuropathic pain. *Nat. Rev. Dis. Primers* 3:17002. 10.1038/nrdp.2017.2 28205574 PMC5371025

[B29] Cornelison L. Woodman S. Durham P. (2020). Inhibition of trigeminal nociception by non-invasive vagus nerve stimulation: Investigating the role of GABAergic and serotonergic pathways in a model of episodic migraine. *Front. Neurol.* 11:146. 10.3389/fneur.2020.00146 32194498 PMC7066071

[B30] Cryan J. O’Riordan K. Cowan C. Sandhu K. Bastiaanssen T. Boehme M. (2019). The microbiota-gut-brain axis. *Physiol. Rev.* 99 1877–2013. 10.1152/physrev.00018.2018 31460832

[B31] Dalile B. Van Oudenhove L. Vervliet B. Verbeke K. (2019). The role of short-chain fatty acids in microbiota-gut-brain communication. *Nat. Rev. Gastroenterol. Hepatol*. 16 461–478. 10.1038/s41575-019-0157-3 31123355

[B32] De Palma G. Shimbori C. Reed D. Yu Y. Rabbia V. Lu J. (2022). Histamine production by the gut microbiota induces visceral hyperalgesia through histamine 4 receptor signaling in mice. *Sci. Transl. Med.* 14:eabj1895. 10.1126/scitranslmed.abj1895 35895832

[B33] de Weerth C. (2017). Do bacteria shape our development? Crosstalk between intestinal microbiota and HPA axis. *Neurosci. Biobehav. Rev*. 83 458–471. 10.1016/j.neubiorev.2017.09.016 28918360

[B34] Deng Y. Zhou M. Wang J. Yao J. Yu J. Liu W. (2021). Involvement of the microbiota-gut-brain axis in chronic restraint stress: Disturbances of the kynurenine metabolic pathway in both the gut and brain. *Gut Microbes* 13 1–16. 10.1080/19490976.2020.1869501 33535879 PMC7872056

[B35] Di Vincenzo F. Del Gaudio A. Petito V. Lopetuso L. Scaldaferri F. (2024). Gut microbiota, intestinal permeability, and systemic inflammation: A narrative review. *Intern. Emerg. Med*. 19 275–293. 10.1007/s11739-023-03374-w 37505311 PMC10954893

[B36] Dicks L. (2022). Gut bacteria and neurotransmitters. *Microorganisms* 10:1838. 10.3390/microorganisms10091838 36144440 PMC9504309

[B37] Dicks L. (2023). Our mental health is determined by an intrinsic interplay between the central nervous system, enteric nerves, and gut microbiota. *Int. J. Mol. Sci.* 25:38. 10.3390/ijms25010038 38203207 PMC10778721

[B38] Eggert T. Bakonyi D. Hummel W. (2014). Enzymatic routes for the synthesis of ursodeoxycholic acid. *J. Biotechnol*. 191 11–21. 10.1016/j.jbiotec.2014.08.006 25131646

[B39] Erny D. Dokalis N. Mezö C. Castoldi A. Mossad O. Staszewski O. (2021). Microbiota-derived acetate enables the metabolic fitness of the brain innate immune system during health and disease. *Cell. Metab*. 33 2260–2276.e7. 10.1016/j.cmet.2021.10.010 34731656

[B40] Faraji N. Payami B. Ebadpour N. Gorji A. (2025). Vagus nerve stimulation and gut microbiota interactions: A novel therapeutic avenue for neuropsychiatric disorders. *Neurosci. Biobehav. Rev.* 169:105990. 10.1016/j.neubiorev.2024.105990 39716559

[B41] Finnerup N. Attal N. Haroutounian S. McNicol E. Baron R. Dworkin R. (2015). Pharmacotherapy for neuropathic pain in adults: A systematic review and meta-analysis. *Lancet Neurol*. 14 162–173. 10.1016/S1474-4422(14)70251-0 25575710 PMC4493167

[B42] Fock E. Parnova R. (2023). Mechanisms of blood-brain barrier protection by microbiota-derived short-chain fatty acids. *Cells* 12:657. 10.3390/cells12040657 36831324 PMC9954192

[B43] Fusco W. Lorenzo M. Cintoni M. Porcari S. Rinninella E. Kaitsas F. (2023). Short-chain fatty-acid-producing bacteria: Key components of the human gut microbiota. *Nutrients* 15:2211. 10.3390/nu15092211 37432351 PMC10180739

[B44] Gao N. Li M. Wang W. Liu Z. Guo Y. (2024). The dual role of TRPV1 in peripheral neuropathic pain: Pain switches caused by its sensitization or desensitization. *Front. Mol. Neurosci.* 17:1400118. 10.3389/fnmol.2024.1400118 39315294 PMC11417043

[B45] Geirnaert A. Calatayud M. Grootaert C. Laukens D. Devriese S. Smagghe G. (2017). Butyrate-producing bacteria supplemented in vitro to Crohn’s disease patient microbiota increased butyrate production and enhanced intestinal epithelial barrier integrity. *Sci. Rep*. 7:11450. 10.1038/s41598-017-11734-8 28904372 PMC5597586

[B46] Gershon M. Tack J. (2007). The serotonin signaling system: From basic understanding to drug development for functional GI disorders. *Gastroenterology* 132 397–414. 10.1053/j.gastro.2006.11.002 17241888

[B47] Gholami-Mahtaj L. Mooziri M. Dehdar K. Abdolsamadi M. Salimi M. Raoufy M. R. (2022). ACC-BLA functional connectivity disruption in allergic inflammation is associated with anxiety. *Sci. Rep*. 12:2731. 10.1038/s41598-022-06748-w 35177766 PMC8854589

[B48] Grüter T. Mohamad N. Rilke N. Blusch A. Sgodzai M. Demir S. (2023). Propionate exerts neuroprotective and neuroregenerative effects in the peripheral nervous system. *Proc. Natl. Acad. Sci. U S A.* 120:e2216941120. 10.1073/pnas.2216941120 36669102 PMC9942889

[B49] Guo B. Zhang M. Hao W. Wang Y. Zhang T. Liu C. (2023). Neuroinflammation mechanisms of neuromodulation therapies for anxiety and depression. *Transl. Psychiatry* 13:5. 10.1038/s41398-022-02297-y 36624089 PMC9829236

[B50] Guo C. Xie S. Chi Z. Zhang J. Liu Y. Zhang L. (2016). Bile Acids control inflammation and metabolic disorder through inhibition of NLRP3 inflammasome. *Immunity* 45 802–816. 10.1016/j.immuni.2016.09.008 27692610

[B51] Guo S. Ma T. Kwok L. Quan K. Li B. Wang H. (2024). Effects of postbiotics on chronic diarrhea in young adults: A randomized, double-blind, placebo-controlled crossover trial assessing clinical symptoms, gut microbiota, and metabolite profiles. *Gut Microbes* 16:2395092. 10.1080/19490976.2024.2395092 39189588 PMC11352714

[B52] Guzior D. Quinn R. (2021). Review: Microbial transformations of human bile acids. *Microbiome* 9:140. 10.1186/s40168-021-01101-1 34127070 PMC8204491

[B53] Herrera G. Castañeda S. Arboleda J. Pérez-Jaramillo J. Patarroyo M. Ramírez J. (2024). Metagenome-assembled genomes (MAGs) suggest an acetate-driven protective role in gut microbiota disrupted by Clostridioides difficile. *Microbiol. Res*. 285:127739. 10.1016/j.micres.2024.127739 38763016

[B54] Hu S. Hu J. Zou F. Liu J. Luo H. Hu D. (2022). P2X7 receptor in inflammation and pain. *Brain Res. Bull.* 187 199–209. 10.1016/j.brainresbull.2022.07.006 35850190

[B55] Huang C. Wang L. Lue J. Chen S. Tsai Y. (2024). Lactobacillus Plantarum intake mitigates neuropathic pain behavior via enhancing macrophage M2 polarization in a rat model of peripheral neuropathy. *Biomed. Pharmacother*. 175:116769. 10.1016/j.biopha.2024.116769 38776678

[B56] Huang F. Wang T. Lan Y. Yang L. Pan W. Zhu Y. (2015). Deletion of mouse FXR gene disturbs multiple neurotransmitter systems and alters neurobehavior. *Front. Behav. Neurosci.* 9:70. 10.3389/fnbeh.2015.00070 25870546 PMC4378301

[B57] Hyland N. Cryan J. F. (2010). A gut feeling about GABA: Focus on GABA(B) receptors. *Front. Pharmacol.* 1:124. 10.3389/fphar.2010.00124 21833169 PMC3153004

[B58] Işık M. Köse F. Özbayer C. Budak Ö Kaya R. K. Erdoğan D. G. (2025). Promising antidepressant potential: The role of Lactobacillus rhamnosus GG in mental health and stress response. *Probiotics Antimicrob. Proteins* 17 5235–5265. 10.1007/s12602-025-10470-0 39962033 PMC12634810

[B59] Janeczko M. Stoll B. Chang X. Guan X. Burrin D. (2007). Extensive gut metabolism limits the intestinal absorption of excessive supplemental dietary glutamate loads in infant pigs. *J. Nutr.* 137 2384–2390. 10.1093/jn/137.11.2384 17951474

[B60] Ji R. Nackley A. Huh Y. Terrando N. Maixner W. (2018). Neuroinflammation and central sensitization in chronic and widespread pain. *Anesthesiology* 129 343–366. 10.1097/ALN.0000000000002130 29462012 PMC6051899

[B61] Jiang H. Zhang X. Yu Z. Zhang Z. Deng M. Zhao J. (2018). Altered gut microbiota profile in patients with generalized anxiety disorder. *J. Psychiatr. Res.* 104 130–136. 10.1016/j.jpsychires.2018.07.007 30029052

[B62] Jiang S. Li H. Zhang L. Mu W. Zhang Y. Chen T. (2025). Generic diagramming platform (GDP): A comprehensive database of high-quality biomedical graphics. *Nucleic Acids Res*. 53 D1670–D1676. 10.1093/nar/gkae973 39470721 PMC11701665

[B63] Jiang X. Ren J. Yu G. Wu W. Chen M. Zhao Y. (2025). Targeting bile-acid metabolism: Nutritional and microbial approaches to alleviate ulcerative colitis. *Nutrients* 17:1174. 10.3390/nu17071174 40218932 PMC11990178

[B64] Jiang Y. Yang J. Wei M. Shou J. Shen S. Yu Z. (2025). Probiotics alleviate painful diabetic neuropathy by modulating the microbiota-gut-nerve axis in rats. *J. Neuroinflammation* 22:30. 10.1186/s12974-025-03352-3 39894793 PMC11789326

[B65] Jones L. Sun E. Martin A. Keating D. (2020). The ever-changing roles of serotonin. *Int J Biochem. Cell. Biol.* 125:105776. 10.1016/j.biocel.2020.105776 32479926

[B66] Joyce S. O’Malley D. (2022). Bile acids, bioactive signalling molecules in interoceptive gut-to-brain communication. *J. Physiol.* 600 2565–2578. 10.1113/JP281727 35413130 PMC9325455

[B67] Jurga A. Rojewska E. Makuch W. Mika J. (2018). Lipopolysaccharide from Rhodobacter sphaeroides (TLR4 antagonist) attenuates hypersensitivity and modulates nociceptive factors. *Pharm. Biol.* 56 275–286. 10.1080/13880209.2018.1457061 29656686 PMC6130482

[B68] Kasubuchi M. Hasegawa S. Hiramatsu T. Ichimura A. Kimura I. (2015). Dietary gut microbial metabolites, short-chain fatty acids, and host metabolic regulation. *Nutrients* 7 2839–2849. 10.3390/nu7042839 25875123 PMC4425176

[B69] Ketel J. Bosch-Bruguera M. Auchter G. Cuntz U. Zipfel S. Enck P. (2024). Gastrointestinal microbiota & symptoms of depression and anxiety in anorexia nervosa-A Re-analysis of the MICROBIAN longitudinal study. *Nutrients* 16:891. 10.3390/nu16060891 38542802 PMC10974745

[B70] Killingsworth J. Sawmiller D. Shytle R. (2020). Propionate and Alzheimer’s disease. *Front. Aging Neurosci.* 12:580001. 10.3389/fnagi.2020.580001 33505301 PMC7831739

[B71] Kopczyñska J. Kowalczyk M. (2024). The potential of short-chain fatty acid epigenetic regulation in chronic low-grade inflammation and obesity. *Front. Immunol*. 15:1380476. 10.3389/fimmu.2024.1380476 38605957 PMC11008232

[B72] Kumar M. Bhatt B. Gusain C. Mahajan N. Bishnoi M. (2024). Sex-specific effects of ketogenic diet on anxiety-like behavior and neuroimmune response in C57Bl/6J mice. *J. Nutr. Biochem.* 127:109591. 10.1016/j.jnutbio.2024.109591 38311044

[B73] Kurhaluk N. Kołodziejska R. Kamiñski P. Tkaczenko H. (2025). Integrative neuroimmune role of the parasympathetic nervous system, vagus nerve and gut microbiota in stress modulation: A narrative review. *Int. J. Mol. Sci*. 26:11706. 10.3390/ijms262311706 41373850 PMC12692660

[B74] Lee Y. Cho Y. Kim J. (2025). The unique role of fluoxetine in alleviating depression and anxiety by regulating gut microbiota and the expression of vagus nerve-mediated serotonin and melanocortin-4 receptors. *Biomed. Pharmacother*. 182:17748. 10.1016/j.biopha.2024.117748 39671722

[B75] Lenin R. Jha K. Gentry J. Shrestha A. Culp E. Vaithianathan T. (2023). Tauroursodeoxycholic acid alleviates endoplasmic reticulum stress-mediated visual deficits in diabetic tie2-TNF transgenic mice via TGR5 signaling. *J. Ocul. Pharmacol. Ther*. 39 159–174. 10.1089/jop.2022.0117 36791327 PMC10081728

[B76] Li K. Wei W. Xu C. Lian X. Bao J. Yang S. (2024). Prebiotic inulin alleviates anxiety and depression-like behavior in alcohol withdrawal mice by modulating the gut microbiota and 5-HT metabolism. *Phytomedicine* 135:156181. 10.1016/j.phymed.2024.156181 39488100

[B77] Lian N. Shen M. Zhang K. Pan J. Jiang Y. Yu Y. (2021). Drinking hydrogen-rich water alleviates chemotherapy-induced neuropathic pain through the regulation of gut microbiota. *J. Pain Res.* 14 681–691. 10.2147/JPR.S288289 33732014 PMC7956896

[B78] Liao J. Hsu C. Chou G. Hsu J. Liong M. Tsai Y. (2019). Lactobacillus paracasei PS23 reduced early-life stress abnormalities in maternal separation mouse model. *Benef. Microbes* 10 425–436. 10.3920/BM2018.0077 30882243

[B79] Ling X. Peng S. Zhong J. Guo L. Xu Y. Jin X. (2022). Effects of Chang-Kang-Fang formula on the microbiota-gut-brain axis in rats with irritable bowel syndrome. *Front. Pharmacol.* 13:778032. 10.3389/fphar.2022.778032 35614949 PMC9125359

[B80] Linnerbauer M. Wheeler M. Quintana F. (2020). Astrocyte crosstalk in CNS inflammation. *Neuron* 108 608–622. 10.1016/j.neuron.2020.08.012 32898475 PMC7704785

[B81] Liu L. Wu Q. Chen Y. Ren H. Zhang Q. Yang H. (2023). Gut microbiota in chronic pain: Novel insights into mechanisms and promising therapeutic strategies. *Int. Immunopharmacol*. 115:109685. 10.1016/j.intimp.2023.109685 37278059

[B82] Liu Q. Yao X. Gao S. Li R. Li B. Yang W. (2020). Role of 5-HT receptors in neuropathic pain: Potential therapeutic implications. *Pharmacol. Res*. 159:104949. 10.1016/j.phrs.2020.104949 32464329

[B83] Liu W. Chuang H. Huang Y. Wu C. Chou G. Wang S. (2016). Alteration of behavior and monoamine levels attributable to Lactobacillus plantarum PS128 in germ-free mice. *Behav. Brain Res.* 298 202–209. 10.1016/j.bbr.2015.10.046 26522841

[B84] Lu G. Zhang S. Wang R. Wu X. Chen Y. Wen Q. (2025). Fecal microbiota transplantation improves bile acid malabsorption in patients with inflammatory bowel disease: Results of microbiota and metabolites from two cohort studies. *BMC Med.* 23:511. 10.1186/s12916-025-04353-y 40890737 PMC12403533

[B85] Lu J. Fan X. Lu L. Yu Y. Markiewicz E. Little J. (2023). Limosilactobacillus reuteri normalizes blood-brain barrier dysfunction and neurodevelopment deficits associated with prenatal exposure to lipopolysaccharide. *Gut Microbes* 15:2178800. 10.1080/19490976.2023.2178800 36799469 PMC9980478

[B86] Lu Y. Zhang Z. Tong L. Zhou X. Liang X. Yi H. (2021). Mechanisms underlying the promotion of 5-hydroxytryptamine secretion in enterochromaffin cells of constipation mice by Bifidobacterium and Lactobacillus. *Neurogastroenterol. Motil.* 33:e14082. 10.1111/nmo.14082 33448546

[B87] Luo X. Yang X. Tan S. Zhang Y. Liu Y. Tian X. (2024). Gut microbiota mediates anxiety-like behaviors induced by chronic infection of Toxoplasma gondii in mice. *Gut Microbes* 16:2391535. 10.1080/19490976.2024.2391535 39182245 PMC11346544

[B88] Lynch C. Clarke G. Cryan J. (2021). Powering up microbiome-microglia interactions. *Cell. Metab.* 33 2097–2099. 10.1016/j.cmet.2021.10.006 34731651

[B89] Ma T. Jin H. Kwok L. Sun Z. Liong M. Zhang H. (2021). Probiotic consumption relieved human stress and anxiety symptoms possibly via modulating the neuroactive potential of the gut microbiota. *Neurobiol. Stress* 14:100294. 10.1016/j.ynstr.2021.100294 33511258 PMC7816019

[B90] Mahdirejei H. Peeri M. Azarbayjani M. Fattahi Masrour F. (2023). Fluoxetine combined with swimming exercise synergistically reduces lipopolysaccharide-induced depressive-like behavior by normalizing the HPA axis and brain inflammation in mice. *Pharmacol. Biochem. Behav.* 232:173640. 10.1016/j.pbb.2023.173640 37741552

[B91] Mayerhofer R. Fröhlich E. Reichmann F. Farzi A. Kogelnik N. Fröhlich E. (2017). Diverse action of lipoteichoic acid and lipopolysaccharide on neuroinflammation, blood-brain barrier disruption, and anxiety in mice. *Brain Behav. Immun*. 60 174–187. 10.1016/j.bbi.2016.10.011 27751870 PMC5419569

[B92] Mirzaei R. Bouzari B. Hosseini-Fard S. Mazaheri M. Ahmadyousefi Y. Abdi M. (2021). Role of microbiota-derived short-chain fatty acids in nervous system disorders. *Biomed. Pharmacother.* 139:111661. 10.1016/j.biopha.2021.111661 34243604

[B93] Mishra S. Jain S. Wang B. Wang S. Miller B. Lee J. (2024). Abnormalities in microbiota/butyrate/FFAR3 signaling in aging gut impair brain function. *JCI Insight* 9:e168443. 10.1172/jci.insight.168443 38329121 PMC10967378

[B94] Mishra S. Karunakar P. Taraphder S. Yadav H. (2020). Free fatty acid receptors 2 and 3 as microbial metabolite sensors to shape host health: Pharmacophysiological view. *Biomedicines* 8:154. 10.3390/biomedicines8060154 32521775 PMC7344995

[B95] Mo X. Guo S. He D. Cheng Q. Yang Y. Wang H. (2025). Lactobacillus reuteri DSM 17,938 ameliorates LPS-induced depression-like and anxiety-like behaviors by modulating gut microbiota and brain metabolic function. *Gut Pathog*. 17:65. 10.1186/s13099-025-00739-8 40841920 PMC12372279

[B96] Mohammad S. Thiemermann C. (2020). Role of metabolic endotoxemia in systemic inflammation and potential interventions. *Front. Immunol*. 11:594150. 10.3389/fimmu.2020.594150 33505393 PMC7829348

[B97] Nankova B. Agarwal R. MacFabe D. La Gamma E. (2014). Enteric bacterial metabolites propionic and butyric acid modulate gene expression, including CREB-dependent catecholaminergic neurotransmission, in PC12 cells–possible relevance to autism spectrum disorders. *PLoS One* 9:e103740. 10.1371/journal.pone.0103740 25170769 PMC4149359

[B98] Nemoto M. Endo T. Minami M. Yoshioka M. Ito H. Saito H. (2001). 5-Hydroxytryptamine (5-HT)-induced depolarization in isolated abdominal vagus nerves in the rat: Involvement of 5-HT3 and 5-HT4 receptors. *Res. Commun. Mol. Pathol. Pharmacol.* 109 217–230. 11758651

[B99] Nøhr M. Pedersen M. Gille A. Egerod K. Engelstoft M. Husted A. (2013). GPR41/FFAR3 and GPR43/FFAR2 as cosensors for short-chain fatty acids in enteroendocrine cells vs FFAR3 in enteric neurons and FFAR2 in enteric leukocytes. *Endocrinology* 154 3552–3564. 10.1210/en.2013-1142 23885020

[B100] Olson C. Vuong H. Yano J. Liang Q. Nusbaum D. Hsiao E. (2018). The gut microbiota mediates the anti-seizure effects of the ketogenic diet. *Cell* 173 1728–1741.e13. 10.1016/j.cell.2018.04.027 29804833 PMC6003870

[B101] Palepu M. Gajula S. Sonti R. Dandekar M. P. (2024). SCFAs supplementation rescues anxiety- and depression-like phenotypes generated by fecal engraftment of treatment-resistant depression rats. *ACS Chem. Neurosci*. 15 1010–1025. 10.1021/acschemneuro.3c00727 38382546

[B102] Park J. Kim C. H. (2021). Regulation of common neurological disorders by gut microbial metabolites. *Exp. Mol. Med.* 53 1821–1833. 10.1038/s12276-021-00703-x 34857900 PMC8741890

[B103] Pezet S. Malcangio M. McMahon S. B. (2002). BDNF: A neuromodulator in nociceptive pathways? *Brain Res. Brain Res. Rev.* 40 240–249. 10.1016/s0165-0173(02)00206-0 12589922

[B104] Pokusaeva K. Johnson C. Luk B. Uribe G. Fu Y. Oezguen N. (2017). GABA-producing Bifidobacterium dentium modulates visceral sensitivity in the intestine. *Neurogastroenterol. Motil.* 29:e12904. 10.1111/nmo.12904 27458085 PMC5195897

[B105] Qu S. Yu Z. Zhou Y. Wang S. Jia M. Chen T. (2024). Gut microbiota modulates neurotransmitter and gut-brain signaling. *Microbiol. Res.* 287:127858. 10.1016/j.micres.2024.127858 39106786

[B106] Rajamanickam G. Lee A. Liao P. (2024). Role of brain derived neurotrophic factor and related therapeutic strategies in central post-stroke pain. *Neurochem. Res*. 49 2303–2318. 10.1007/s11064-024-04175-z 38856889

[B107] Ramakrishna C. Corleto J. Ruegger P. Logan G. Peacock B. Mendonca S. (2019). Dominant role of the gut microbiota in chemotherapy induced neuropathic pain. *Sci. Rep*. 9:20324. 10.1038/s41598-019-56832-x 31889131 PMC6937259

[B108] Ridlon J. Bajaj J. (2015). The human gut sterolbiome: Bile acid-microbiome endocrine aspects and therapeutics. *Acta Pharm. Sin. B* 5 99–105. 10.1016/j.apsb.2015.01.006 26579434 PMC4629220

[B109] Riehl L. Fürst J. Kress M. Rykalo N. (2023). The importance of the gut microbiome and its signals for a healthy nervous system and the multifaceted mechanisms of neuropsychiatric disorders. *Front. Neurosci*. 17:1302957. 10.3389/fnins.2023.1302957 38249593 PMC10797776

[B110] Romanazzi T. Zanella D. Bhatt M. Di Iacovo A. Galli A. Bossi E. (2023). Bile acid interactions with neurotransmitter transporters. *Front. Cell. Neurosci.* 17:1161930. 10.3389/fncel.2023.1161930 37180953 PMC10169653

[B111] Rooks M. Garrett W. (2016). Gut microbiota, metabolites and host immunity. *Nat. Rev. Immunol*. 16 341–352. 10.1038/nri.2016.42 27231050 PMC5541232

[B112] Royo F. Tames H. Bordanaba-Florit G. Cabrera D. Azparren-Angulo M. Garcia-Vallicrosa C. (2023). Orally administered bifidobacterium adolescentis diminishes serum glutamate concentration in mice. *Microbiol. Spectr*. 11:e0506322. 10.1128/spectrum.05063-22 37347184 PMC10433951

[B113] Rusch J. Layden B. Dugas L. (2023). Signalling cognition: The gut microbiota and hypothalamic-pituitary-adrenal axis. *Front. Endocrinol*. 14:1130689. 10.3389/fendo.2023.1130689 37404311 PMC10316519

[B114] Sagalajev B. Bourbia N. Beloushko E. Wei H. Pertovaara A. (2015). Bidirectional amygdaloid control of neuropathic hypersensitivity mediated by descending serotonergic pathways acting on spinal 5-HT3 and 5-HT1A receptors. *Behav. Brain Res*. 282 14–24. 10.1016/j.bbr.2014.12.052 25557801

[B115] Salminen A. (2023). Activation of aryl hydrocarbon receptor (AhR) in Alzheimer’s disease: Role of tryptophan metabolites generated by gut host-microbiota. *J. Mol. Med.* 101 201–222. 10.1007/s00109-023-02289-5 36757399 PMC10036442

[B116] Sanz-Salvador L. Andrés-Borderia A. Ferrer-Montiel A. Planells-Cases R. (2012). Agonist- and Ca2+-dependent desensitization of TRPV1 channel targets the receptor to lysosomes for degradation. *J. Biol. Chem*. 287 19462–19471. 10.1074/jbc.M111.289751 22493457 PMC3365984

[B117] Shi W. Yao Y. Liang Y. Lei J. Feng S. Zhang Z. (2025). Activation of TGR5 in the injured nerve site according to a prevention protocol mitigates partial sciatic nerve ligation-induced neuropathic pain by alleviating neuroinflammation. *Pain* 166 1296–1313. 10.1097/j.pain.0000000000003460 39450924 PMC12067609

[B118] Shin H. Jo B. Lee C. Lee K. Namgung U. (2019). Hippocampal activation of 5-HT1B receptors and BDNF production by vagus nerve stimulation in rats under chronic restraint stress. *Eur. J. Neurosci.* 50 1820–1830. 10.1111/ejn.14368 30735600

[B119] Shu L. Ding Y. Xue Q. Cai W. Deng H. (2023). Direct and indirect effects of pathogenic bacteria on the integrity of intestinal barrier. *Therap. Adv. Gastroenterol*. 16:17562848231176427. 10.1177/17562848231176427 37274298 PMC10233627

[B120] Šimić G. Tkalčić M. Vukić V. Mulc D. Španić E. Šagud M. (2021). Understanding emotions: Origins and roles of the amygdala. *Biomolecules* 11:823. 10.3390/biom11060823 34072960 PMC8228195

[B121] Singh N. Gurav A. Sivaprakasam S. Brady E. Padia R. Shi H. (2014). Activation of Gpr109a, receptor for niacin and the commensal metabolite butyrate, suppresses colonic inflammation and carcinogenesis. *Immunity* 40 128–139. 10.1016/j.immuni.2013.12.007 24412617 PMC4305274

[B122] Singh V. Lee G. Son H. Koh H. Kim E. Unno T. (2022). Butyrate producers, the sentinel of gut: Their intestinal significance with and beyond butyrate, and prospective use as microbial therapeutics. *Front. Microbiol.* 13:1103836. 10.3389/fmicb.2022.1103836 36713166 PMC9877435

[B123] Smith P. A. (2024). BDNF in neuropathic pain; the culprit that cannot be apprehended. *Neuroscience* 543 49–64. 10.1016/j.neuroscience.2024.02.020 38417539

[B124] Strandwitz P. Kim K. Terekhova D. Liu J. Sharma A. Levering J. (2019). GABA-modulating bacteria of the human gut microbiota. *Nat. Microbiol.* 4 396–403. 10.1038/s41564-018-0307-3 30531975 PMC6384127

[B125] Sun N. Hu H. Wang F. Li L. Zhu W. Shen Y. (2021). Antibiotic-induced microbiome depletion in adult mice disrupts blood-brain barrier and facilitates brain infiltration of monocytes after bone-marrow transplantation. *Brain Behav. Immun*. 92 102–114. 10.1016/j.bbi.2020.11.032 33242652

[B126] Takanaga H. Ohtsuki S. Hosoya K. Terasaki T. (2001). GAT2/BGT-1 as a system responsible for the transport of gamma-aminobutyric acid at the mouse blood-brain barrier. *J. Cereb. Blood Flow. Metab.* 21 1232–1239. 10.1097/00004647-200110000-00012 11598501

[B127] Tao Y. Zhou H. Li Z. Wu H. Wu F. Miao Z. (2024). TGR5 deficiency-induced anxiety and depression-like behaviors: The role of gut microbiota dysbiosis. *J. Affect. Disord.* 344 219–232. 10.1016/j.jad.2023.10.072 37839469

[B128] Tominaga M. Caterina M. Malmberg A. Rosen T. Gilbert H. Skinner K. (1998). The cloned capsaicin receptor integrates multiple pain-producing stimuli. *Neuron* 21 531–543. 10.1016/s0896-6273(00)80564-4 9768840

[B129] Tsukuda N. Yahagi K. Hara T. Watanabe Y. Matsumoto H. Mori H. (2021). Key bacterial taxa and metabolic pathways affecting gut short-chain fatty acid profiles in early life. *ISME J.* 15 2574–2590. 10.1038/s41396-021-00937-7 33723382 PMC8397723

[B130] van Hecke O. Austin S. Khan R. Smith B. Torrance N. (2014). Neuropathic pain in the general population: A systematic review of epidemiological studies. *Pain* 155 654–662. 10.1016/j.pain.2013.11.013 24291734

[B131] Wang J. Zhu N. Su X. Gao Y. Yang R. (2023). Gut-microbiota-derived metabolites maintain gut and systemic immune homeostasis. *Cells* 12:793. 10.3390/cells12050793 36899929 PMC10000530

[B132] Wang X. Duan C. Li Y. Lu H. Guo K. Ge X. (2022). Sodium butyrate reduces overnutrition-induced microglial activation and hypothalamic inflammation. *Int. Immunopharmacol*. 111:109083. 10.1016/j.intimp.2022.109083 35917736

[B133] Wang Y. Han Q. Gong W. Pan D. Wang L. Hu W. (2018). Microglial activation mediates chronic mild stress-induced depressive- and anxiety-like behavior in adult rats. *J. Neuroinflammation* 15:21. 10.1186/s12974-018-1054-3 29343269 PMC5773028

[B134] Wang Y. Zhang X. Yao Y. Hu S. Wang W. Wang D. (2024). Inferior social hierarchy is vulnerable to anxiety-like behavior in chronic pain mice: Potential role of gut microbiota and metabolites. *Neurobiol. Dis.* 191:106402. 10.1016/j.nbd.2024.106402 38184015

[B135] Wegner A. Elsenbruch S. Maluck J. Grigoleit J. Engler H. Jäger M. (2014). Inflammation-induced hyperalgesia: Effects of timing, dosage, and negative affect on somatic pain sensitivity in human experimental endotoxemia. *Brain Behav. Immun.* 41 46–54. 10.1016/j.bbi.2014.05.001 24814500

[B136] Wei H. Yu C. Zhang C. Ren Y. Guo L. Wang T. (2023). Butyrate ameliorates chronic alcoholic central nervous damage by suppressing microglia-mediated neuroinflammation and modulating the microbiome-gut-brain axis. *Biomed. Pharmacother*. 160:114308. 10.1016/j.biopha.2023.114308 36709599

[B137] Weiss G. A. Hennet T. (2017). Mechanisms and consequences of intestinal dysbiosis. *Cell. Mol. Life Sci.* 74 2959–2977. 10.1007/s00109-023-02289-5 28352996 PMC11107543

[B138] Weng H. Deng L. Wang T. (2024). Humid heat environment causes anxiety-like disorder via impairing gut microbiota and bile acid metabolism in mice. *Nat. Commun.* 15:5697. 10.1038/s41467-024-49972-w 38972900 PMC11228019

[B139] Włodarczyk A. Cubała W. Wielewicka A. (2020). Ketogenic diet: A dietary modification as an anxiolytic approach? *Nutrients* 12:3822. 10.3390/nu12123822 33327540 PMC7765029

[B140] Woodburn S. Bollinger J. Wohleb E. (2021). The semantics of microglia activation: Neuroinflammation, homeostasis, and stress. *J. Neuroinflammation* 18:258. 10.1186/s12974-021-02309-6 34742308 PMC8571840

[B141] Wu Y. Qiu Y. Su M. Wang L. Gong Q. Wei X. (2023). Activation of the bile acid receptors TGR5 and FXR in the spinal dorsal horn alleviates neuropathic pain. *CNS Neurosci. Ther.* 29 1981–1998. 10.1111/cns.14154 36880297 PMC10324360

[B142] Wu Y. Zhang Y. Xie B. Abdelgawad A. Chen X. Han M. (2021). RhANP attenuates endotoxin-derived cognitive dysfunction through subdiaphragmatic vagus nerve-mediated gut microbiota-brain axis. *J. Neuroinflammation* 18:300. 10.1186/s12974-021-02356-z 34949194 PMC8697447

[B143] Yang W. Cong Y. (2021). Gut microbiota-derived metabolites in the regulation of host immune responses and immune-related inflammatory diseases. *Cell. Mol. Immunol*. 18 866–877. 10.1038/s41423-021-00661-4 33707689 PMC8115644

[B144] Yano J. Yu K. Donaldson G. Shastri G. Ann P. Ma L. (2015). Indigenous bacteria from the gut microbiota regulate host serotonin biosynthesis. *Cell* 161 264–276. 10.1016/j.cell.2015.02.047 25860609 PMC4393509

[B145] Yu K. Hsiao E. (2021). Roles for the gut microbiota in regulating neuronal feeding circuits. *J. Clin. Invest.* 131:e143772. 10.1172/JCI143772 33998595 PMC8121503

[B146] Yu W. Xiao Y. Jayaraman A. Yen Y. Lee H. Pettersson S. (2025). Microbial metabolites tune amygdala neuronal hyperexcitability and anxiety-linked behaviors. *EMBO Mol. Med*. 17 249–264. 10.1038/s44321-024-00179-y 39910348 PMC11821874

[B147] Yuan X. Chen B. Duan Z. Xia Z. Ding Y. Chen T. (2021). Depression and anxiety in patients with active ulcerative colitis: Crosstalk of gut microbiota, metabolomics and proteomics. *Gut Microbes* 13:1987779. 10.1080/19490976.2021.1987779 34806521 PMC8632339

[B148] Zhang H. Wang Z. Wang G. Song X. Qian Y. Liao Z. (2023). Understanding the connection between gut homeostasis and psychological stress. *J. Nutr*. 153 924–939. 10.1016/j.tjnut.2023.01.026 36806451

[B149] Zhang J. Xian H. Zhao R. Luo C. Xie R. Tian T. (2023). Brachial plexus avulsion induced changes in gut microbiota promotes pain related anxiety-like behavior in mice. *Front. Neurol.* 14:1084494. 10.3389/fneur.2023.1084494 36846129 PMC9944865

[B150] Zhang K. Chen L. Yang J. Liu J. Li J. Liu Y. (2023). Gut microbiota-derived short-chain fatty acids ameliorate methamphetamine-induced depression- and anxiety-like behaviors in a Sigmar-1 receptor-dependent manner. *Acta Pharm. Sin. B* 13 4801–4822. 10.1016/j.apsb.2023.09.010 38045052 PMC10692394

[B151] Zhang N. Gao X. Li D. Xu L. Zhou G. Xu M. (2024). Sleep deprivation-induced anxiety-like behaviors are associated with alterations in the gut microbiota and metabolites. *Microbiol. Spectr*. 12:e0143723. 10.1128/spectrum.01437-23 38421192 PMC10986621

[B152] Zhang P. Zhang C. Zheng B. Liu Y. Zhang D. Xiao H. (2025). The brain-gut mechanism of postherpetic neuralgia: A mini-review. *Front. Neurol.* 16:1535136. 10.3389/fneur.2025.1535136 40129863 PMC11932021

[B153] Zheng Z. Tu J. Li X. Hua Q. Liu W. Liu Y. (2021). Neuroinflammation induces anxiety- and depressive-like behavior by modulating neuronal plasticity in the basolateral amygdala. *Brain Behav. Immun*. 91 505–518. 10.1016/j.bbi.2020.11.007 33161163

[B154] Zhong S. Liu F. Giniatullin R. Jolkkonen J. Li Y. Zhou Z. (2023). Blockade of CCR5 suppresses paclitaxel-induced peripheral neuropathic pain caused by increased deoxycholic acid. *Cell. Rep.* 42:113386. 10.1016/j.celrep.2023.113386 37948181

[B155] Zhu J. Zhong Z. Shi L. Huang L. Lin C. He Y. (2024). Gut microbiota mediate early life stress-induced social dysfunction and anxiety-like behaviors by impairing amino acid transport at the gut. *Gut Microbes* 16:2401939. 10.1080/19490976.2024.2401939 39259834 PMC11404583

[B156] Zhuang M. Zhang X. Cai J. (2024). Microbiota-gut-brain axis: Interplay between microbiota, barrier function and lymphatic system. *Gut Microbes* 16 2387800. 10.1080/19490976.2024.2387800 39182226 PMC11346530

[B157] Zou Q. Han S. Liang J. Yan G. Wang Q. Wang Y. (2024). Alleviating effect of vagus nerve cutting in *Salmonella*-induced gut infections and anxiety-like behavior via enhancing microbiota-derived GABA. *Brain Behav. Immun*. 119 607–620. 10.1016/j.bbi.2024.04.034 38663772

